# Neurostimulation for Intractable Chronic Pain

**DOI:** 10.3390/brainsci9020023

**Published:** 2019-01-24

**Authors:** Timothy R. Deer, Sameer Jain, Corey Hunter, Krishnan Chakravarthy

**Affiliations:** 1 Spine and Nerve Center of the Virginias, Charleston, VA 25301, USA; 2 Pain Treatment Centers of America, Little Rock, AR 72205, USA; paindocsj@gmail.com; 3 Ainsworth Institute of Pain Management, New York, NY 10022, USA; chunter@ainpain.com; 4 Department of Anesthesiology and Pain Medicine, University of California San Diego Health Sciences, San Diego, CA 92037, USA; kvchakravarthy@ucsd.edu

**Keywords:** neuromodulation, neurostimulation, spinal cord stimulation, dorsal root ganglion stimulation, peripheral nerve stimulation, chronic pain

## Abstract

The field of neuromodulation has seen unprecedented growth over the course of the last decade with novel waveforms, hardware advancements, and novel chronic pain indications. We present here an updated review on spinal cord stimulation, dorsal root ganglion stimulation, and peripheral nerve stimulation. We focus on mechanisms of action, clinical indications, and future areas of research. We also present current drawbacks with current stimulation technology and suggest areas of future advancements. Given the current shortage of viable treatment options using a pharmacological based approach and conservative interventional therapies, neuromodulation presents an interesting area of growth and development for the interventional pain field and provides current and future practitioners a fresh outlook with regards to its place in the chronic pain treatment paradigm.

## 1. Introduction

Pain is an unpleasant sensory and emotional experience that involves complex processes of neuronal signaling in the peripheral nervous system (PNS) and the central nervous system (CNS). Chronic pain may be defined as pain persistent for more than 3–6 months [1]. For decades, chronic pain conditions continue to pose an immense burden on the economy and society in the form of healthcare expenditures and years lived with disability (YLD). Lower back pain alone has been the leading cause of YLDs for the past three decades [2]. In the United States, the healthcare expenditure secondary to chronic pain conditions in the year 2010 was estimated to be $560–$635 billion dollars [3]. This cost was more than the combined expenditure on heart diseases and diabetes mellitus. Globally, 10% of adults are diagnosed with chronic pain conditions each year [4]. Considering the vast amount of suffering caused by chronic pain conditions, international resolutions were made to make access to adequate pain therapy a human right [5,6]. Unfortunately, chronic pain has been known to be notoriously resistant to conventional medical management (CMM) [7,8]. This drove physicians to resort to using opioid therapy to manage chronic pain, inadvertently leading to what we now known as the “Opioid crisis”. There have been an increasing number of deaths involving the overuse of prescription opioids [9]. Unfortunately, this upward trend has continued and remains a major cause of morbidity and mortality. Physicians are now on the constant lookout for opioid sparing therapies to manage chronic pain, and neuromodulation may be the answer. Neuromodulation is the process of inhibition, stimulation, modification, or therapeutic alteration of activity in the CNS, PNS, or the autonomic nervous system (ANS), with the use of electricity. In this review, we will highlight some of the important targets for neuromodulation therapy, their mechanism of action, and the evidence to support their use in the treatment of chronic intractable pain conditions.

## 2. Materials and Methods

The authors performed a search of the current literature using PubMed, Google scholar, Cochrane, Embase, and Medline database. In addition, the review of current scientific meetings, proceedings, and regulatory approvals were used to focus on modern advancements in the field. We selected and cited the major peer-reviewed publications supporting the use of neuromodulation for the management of various painful conditions.

### 2.1. Spinal Cord Stimulation (SCS)

The most simplistic description of Spinal Cord Stimulation (SCS) may be the application of electricity to the dorsal columns of the spinal cord to modulate/manipulate the pain signals carried by the ascending pain pathways to the brain, and hence is also known as dorsal column stimulation (DCS). The concept of SCS derives its inspiration from the landmark “Gate control theory of Pain” proposed by Melzack and Wall in 1965 [10]. This theory postulated the existence of a “Gate” in the dorsal horn of the spinal cord controlling the traffic of neuronal impulses from the sensory afferent neurons to the higher centers in the brain responsible for pain perception. Aβ fibers (responsible for carrying the non-nociceptive stimuli) and C fibers (responsible for carrying the painful stimuli) form synapses with the projection neurons of the spinothalamic tract on the dorsal horn of the spinal cord, which are responsible for the transmission of pain signals to the brain. According to the “gate control theory”, stimulation of the Aβ fibers in the same region as the C fibers can result in the closure of the “gate”, and thus resulting in blocking the transmission of pain impulses. In the spinal cord, these fibers are conveniently segregated from the motor fibers and are in an accessible location, making the dorsal columns a desirable target for stimulation. Based on this theory, Shealy et al. implanted the first dorsal column stimulator in 1967 for the treatment of pain [11]. However, several decades of research has shown that the mechanism of SCS in the treatment of pain is much more complex and continues to elude us.

### 2.2. Parameters of Stimulation

In order to understand the new stimulation paradigms and their mechanisms of action, it is critical to get a better understanding of how the delivery of charge to the spinal cord is manipulated. The three main parameters of stimulation include amplitude, pulse width, and frequency. The basic unit of electrical stimulation in neuromodulation is the “pulse”, which consists of a sustained delivery of a specific amount of current amplitude (measured in milliamperes, mA) for a specific amount of time (pulse width, measured in microseconds, μs). The amount of charge delivered with each pulse is equivalent to the product of amplitude and pulse width, whereas, frequency determines the number of pulses delivered per second. Thus, alteration in the values of these parameters determines the amount of current (amount of charge delivered per second) that is delivered to the neurons. Therefore, narrow pulse widths require high amplitudes to activate the neuron or axon whereas wider pulse widths need lower amplitudes. The amount of charge needed to activate an axon in vivo depends upon the size, myelination, and the distance from the stimulation source. Primarily amplitude with some contribution from pulse width determines the number of fibers recruited and results in a perceived increase or decrease in the intensity and/or area of paresthesia sensation. Frequency of stimulation influences how often a neuron fires in response to a stimulus.

### 2.3. SCS Waveforms and Their Mechanisms of Action

#### 2.3.1. Conventional/Tonic SCS

Conventional/tonic stimulation was the only stimulation paradigm available until few years ago and continues to be the most frequently used in clinical practice. This stimulation paradigm is characterized by low frequency (40–100 Hz), high amplitude (3.6–8.5 mA), and pulse widths ranging between 300–600 μs. It results in the delivery of “high charge” per pulse resulting in the perceived “paresthesias” by the patient. This SCS program has demonstrated superiority over conventional medical management (CMM) strategies in the treatment of several neuropathic (e.g., complex regional pain syndrome (CRPS), diabetic neuropathy, neuropathic limb pain, etc.) and mixed neuropathic (e.g., failed back surgery syndrome (FBSS)) chronic pain conditions (Table 1).

Even though “gate control theory” formed the basis of spinal cord stimulation, but it was primitive and failed to explain why SCS was ineffective in the treatment of acute nociceptive pain. Several theories have been proposed since then explaining the mechanism of action of SCS. Hyperexcitability of the wide-dynamic range (WDR) neurons in the dorsal horn (DH) of the spinal cord has been demonstrated in neuropathic pain states [12]. In animal models, SCS frequencies around 50 Hz have shown to induce release of inhibitory neurotransmitters like GABA resulting in inhibition of the WDR hyperexcitability [13,14]. It has also been suggested that SCS results in release of acetylcholine and its action on muscarinic M4 receptors may be responsible for its analgesic effects [15]. Furthermore, evidence indicates that the pain reduction with SCS may be secondary to stimulation-induced release of serotonin, adenosine, and noradrenaline [16]. Recent evidence suggests the involvement of supraspinal circuitry in mediating the analgesic effects of SCS [17,18]. However, the exact mechanism for the analgesic effects of SCS is still not clear.

#### 2.3.2. High Frequency (HF) SCS

Recent clinical investigations have emphasized the importance of the way energy is delivered to the neural structures in neuromodulation therapies [25]. This has led the scientists in the last few years to focus on the development of new waveforms and stimulation paradigms. High frequency (10 khz) stimulation with a pulse width at 30 μs and amplitude ranging between 1–5 mA is among the most recent developments made on that front [26]. This stimulation therapy has shown superiority over conventional/tonic stimulation in the treatment of chronic low back pain and improving quality of life in a randomized controlled trial (RCT) (Table 2). However, there is still no evidence to support the use of HF SCS over conventional SCS stimulation in the treatment of chronic neuropathic limb pain.

The mechanisms by which HF stimulation results in analgesia are not fully understood, but several working hypotheses have been proposed. One of the theories is that it induces a depolarization blockade (a local reversible block), where propagating action potentials are blocked by HF stimulation [27]. Another hypothesis is that HF stimulation can induce a desynchronization of neural signals from clusters of neurons firing in synchrony. This results in pseudospontaneous or stochastic neural activity, where firing becomes individualized such that each unit is firing at its own rate and pattern [28,29]. “Membrane integration” has also been suggested as a possible mechanism of action for HF SCS, where multiple impulses reaching a neuron within a certain time frame may depolarize it and fire an action potential although every individual impulse is insufficient [30].

#### 2.3.3. Burst SCS

This novel SCS waveform (series of five 1000 μs pulses delivered at 500 Hz followed by a repolarization pulse, with each series repeated at 40 Hz) is another SCS paradigm that has proven superior to conventional SCS in the treatment of lumbosacral component of pain (Table 3). This waveform is reported to mimic the firing patterns of endogenous neurons responsible for encoding aspects of pain signaling in the thalamus [33,34,35]. De ridder et al., on the basis of “source localized EEG” findings also postulated that burst SCS, via modulation of the medial spinothalamic pathway, could activate cortical areas involved in the modulation of pain perception [36]. Thus, making it capable of engaging both spinal and supraspinal pathways in both an anterograde and retrograde fashion as well as those medial and lateral supraspinal pathways. Another hypothesis is that burst firing may be capable of disrupting the synchronous burst firing of the high threshold fibers and inhibiting the activation directly related to pain perception [37]. Even though burst SCS has been shown to be more effective than the tonic SCS stimulation in the treatment of mixed neuropathic pain syndromes like FBSS [38,39], there is not enough evidence to support its superiority in the treatment of pure neuropathic pain states like diabetic neuropathy, CRPS, etc. A prospective observational study was conducted to compare burst SCS vs. HF SCS on a small cohort of 14 FBSS patients who underwent trials with burst (*n* = 8) and HF SCS (*n* = 6) [40,41]. Even though no significant difference was found in the effectiveness to treat the low back pain, burst SCS was slightly superior (not statistically significant) to HF SCS in treating the leg pain component.

### 2.4. Closed-Loop Spinal Cord Stimulation

Even though SCS therapy has numerous proven benefits, it does seem to have certain flaws. One of the major issues with conventional SCS (open-loop) systems is the need for manual adjustment of stimulation current to maintain coverage during postural changes. The position of SCS electrodes in relation to the dorsal column of the spinal cord is dynamic and varies with postural changes [43,44]. This results in unwanted side effects and sometimes loss of therapy. For example, a decrease in the distance between the electrodes and the spinal cord may result in activation of unwanted fibers, which may result in unwanted or uncomfortable paresthesias, muscle twitching, and cramping. Conversely, an increase in the distance between the spinal cord and the SCS electrodes may result in loss of therapy. Loss of therapy/efficacy with spinal cord stimulation has been a concern among pain physicians for a long period of time. Previous studies have demonstrated that effective pain control with SCS decreases over time [45,46,47]. A prospective study demonstrated a linear increase in VAS scores after one and two years of follow up (*p* = 0.03). However, VAS scores were still significantly lower than pre-SCS therapy. A systematic literature review demonstrated successful pain relief in 62% patients at one year with SCS therapy, whereas the success rate dropped to 53% and 35% patients at five and 10 year follow up respectively [48]. Some of the possible causes that have been speculated for this loss of therapy are progression in the underlying disease, change in paresthesia coverage, device migration/malfunction, changes in microenvironment of the electrode leading to high impedances [49].

Closed-loop SCS was developed to neutralize the side effects encountered with postural changes. This stimulation therapy measures individual evoked compound action potential (ECAP) and uses them as a feedback control mechanism to automatically maintain desired dorsal column fiber recruitment levels. The ECAP amplitude at which patient experiences optimal pain relief is set as the reference and the feedback algorithm alters the input current to maintain it constant. Russo et al. published the preliminary results of a prospective, multicenter, single-arm study showing effectiveness and safety of the closed-loop SCS system in the treatment of leg and low back pain [50]. In this study, 51 patients with chronic low back and leg pain underwent a trial with closed-loop SCS system. Thirty-six patients later underwent permanent implantation and were followed for six months. Significant reductions (≥80%) in pain were observed in 70.4% (back pain) and 56.5% (leg pain) patients at the 3-month interval, and 64.3% (back pain) and 60.9% (leg pain) patients at 6-month follow up. Statistically significant improvements in mean BPI (Brief pain inventory), EQ-5D-5L, ODI (Oswestry disability index), and PSQI (Pittsburgh sleep quality index) were also observed at both time points.

### 2.5. Dorsal Root Ganglion (DRG) Stimulation

While the utility and efficacy of traditional SCS is well-established in the literature, the therapy is not without its shortcomings. These deficiencies range from paresthesias in unwanted areas and waning relief over time to position-related changes in stimulation intensity and the inability to capture focal areas like the foot and pelvic region [51,52,53,54,55,56,57]. Perhaps one of the most pressing concerns surrounding the traditional SCS was its inability to provide sustained pain relief in patients with chronic, focal neuropathic pain despite being considered by many to be the “treatment of choice” for such conditions. Long-term data from a prospective study suggested that treating CRPS with SCS and physical therapy may be no better than physical therapy alone after 2-years [58]. These shortcomings led scientists and clinicians to look for new targets within the central nervous system as means to improve upon the therapy that is neuromodulation; one such target was the DRG.

The DRG was long thought of as a passive neural structure that acted solely as a support structure, facilitating communication between peripheral and central nervous systems [59]. The idea that it played any relevant role in the development or maintenance of chronic neuropathic pain had not been elucidated until recently. Current evidence suggests that the DRG, itself, is directly responsible for the development of neuropathic pain through “hyperexcitability” and “spontaneous ectopic firing” of those neurons contained within the DRG [59,60], two processes mainly responsible for central sensitization and allodynia (the hallmarks of CRPS). When one also takes into account the DRG’s role in the modulation of sensory processing and nociceptive pain as well as predictable anatomical location and scarcity of CSF that would otherwise deflect energy [61,62,63], the DRG appeared to be an ideal target for stimulation that could potentially bridge the gap for neurostimulation as a whole.

### 2.6. Physiology

When a peripheral nerve become injured on inflamed, there are a number of changes that occur within the actual DRG:

Gene expression [64]

Microglial cells [65]

Ion channels & current [59]

Chemokines [59]

Ectopic Firing [59,60]

Hyperexcitability [59,60]

Even more interesting is the role the DRG plays in the filtering of transmissions from the peripheral nervous system into the central. The cell bodies of the neurons located with the DRG possess a t-junction that give them the ability to filter action potentials and pool stimuli from the periphery until a certain threshold is achieved before opening up and allowing the signal into the central nervous system [66,67,68].

Taking into account the sheer variety of relevant processes now known occur at the level of the DRG, targeting it for neuromodulation appeared to be a logical conclusion. DRG stimulation is believed to impact pain by applying a variety of effects the processes thought to not only develop chronic pain, but maintain it [69,70,71]:

- Activation of supraspinal centers and the deactivation of hyperexcitability of wide-dynamic range (WDR) neurons located within the dorsal horn;
- Upstream/downstream effects causing stabilization of peripheral nociceptor sensitization, vasodilation, release of neuromodulators in the dorsal horn, and activation of WDR neurons;
- Theorized normalization of gene expression within the DRG and spinal cord;
- Augmentation of T-junction “low pass filter” thus reducing propagation of action potential to the dorsal horn;
- Decreased hyperexcitability of neurons within the DRG by down regulation of abnormal; Na^+^ channels, up-regulation of K^+^ channels and restoration of normal calcium flow;
- Stabilizing microglia releasing cytokines (TNF-α, chemokines, nerve growth factors, interleukins, interferons, etc.).

### 2.7. Evidence for Efficacy

The primary indication for DRGs is focal neuropathic pain, namely CRPS. The early pilot studies by Deer, Grigsby, and Liem not only proved the concept that stimulating the DRG was viable, but also that superior levels of pain relief not typically attainable with conventional SCS were reported (Table 4). In 2012, Deer et al. reported on a prospective study of 10 patients trialed with DRG for 3–7 days; complaints included discogenic pain, low back pain with radicular symptoms, DPPN, PHN (Post-herpetic neuralgia) and neuropathic chest wall pain [56]. This pilot study showed a 70% reduction in pain in the majority of patients with commensurate decrease in opioid consumption.

In 2013, Liem et al. reported on the results of a prospective, 1-year study of 32 patients treated with DRGS [72]. The patients in this study included CRPS, Failed Back Surgery Syndrome (FBSS), chronic post-surgical pain, PHN, spinal stenosis, discogenic pain and radicular pain. Overall pain reduction was 56% with 52% of the subjects reporting >50% improvement in pain. More importantly, was the 80% reduction in foot pain, an area of the body that traditionally been difficult to treat with SCS. Additionally, the authors reported that the patients denied posture-dependent fluctuations in paresthesias commonly associated with SCS.

In 2017, Deer et al. reported the results of the ACCURATE study, a multicenter, randomized, controlled trial of 152 subjects with CRPS treated with DRGS and followed out to 1-year (control group received traditional dorsal column SCS) [73]. The study showed that DRGS was statistically superior to traditional SCS with 74.2% of the DRGS group reporting 50% or more pain relief at 1-year, compared to the control group’s 53%. In addition, patients treated with DRGS reported 81.4%–86% decrease in VAS compared to 48.1%–70.2% decreases in the control group.

Since the inception DRGS, a number of manuscripts have been published on a variety of unique and novel uses that have proven truly groundbreaking, not only for neuromodulation, but the field pain medicine as a whole. Syndromes that had proven to be recalcitrant to most well-accepted pain treatments, including SCS, now had published evidence showing they could potentially be treated with DRG stimulation:

- Post-herniorrhaphy neuralgia [74,75]
- Post-amputee pain and phantom limb pain [76,77]
- Post surgical chest wall pain (i.e., post-mastectomy & post-thoractomy pain) [78,79,80]
- Chronic pelvic pain [57]
- Knee pain after total joint arthroplasty [74,81]
- Post-herpetic neuralgia [56,72,74]
- Diabetic peripheral neuropathy [23,82,83,84]

In 2018, Deer et al. published a “Best Practices” manuscript on the use of DRGS, along with a grading of the available evidence as well as recommendations on its use for various indications [85]. Aside from CRPS (which has Level-I evidence to support) most the other indications had varying degrees of Level-II evidence with recommendation grades ranging between A to B (extremely recommendable to recommendable).

### 2.8. Peripheral Nerve Stimulation

An area of growing interest in the field of neuromodulation has been peripheral nerve stimulation (PNS). With the ability to limit the amount of energy dispersion by using focalized current this area of therapy provides an unprecedented opportunity to treat a multitude of chronic pain disorders. In 1999, the first peripheral nerve leads were placed percutaneously to manage intractable headaches [86]. This has been expanded to include modulation of visceral, neuropathic, cardiac, abdominal, back, and facial pain. Though there are many studies to deduce PNS mechanism of action that validate the Wall and Melzack gate control theory, it has been postulated that PNS is used as a method of orthodromic stimulation of non-nociceptive Aβ nerve fibers. Activation of these fibers results in excitation of respective dorsal horn inter-neurons that are involved in processing and transmitting nociceptive information via peripheral Aβ and C nerve fibers. Thus, non-painful stimulation of the peripheral nerve territory results in decreased pain signals [87]. Studies have suggested an acute modulation of the local microenvironment with down-regulation of neurotransmitters and endorphins in addition to local inflammatory mediators may also be a critical piece on how PNS may be effective in treating chronic pain. Other potential methods of pain modulation could result from reducing ectopic discharges in addition to reducing Wallerian degeneration.

### 2.9. Summary of Clinical Indications

There is growing evidence of the use of peripheral nerve stimulation in a variety of clinical indications that include plexus injuries, focal mononeuropathy, post-amputation pain, back pain, sacroiliac joint pain, headache, facial pain, arm and limb pain. Prior studies have shown that there are good outcomes from PNS on median, ulnar, sciatic, ilioinguinal, and genito-femoral nerves [88,89,90,91,92]. Specific data also supports use of PNS following stimulation of brachial plexus and lumbar plexus with reduction in neuropathic pain, allodynia and restoration of normal tactile sensation following respective plexus injuries [93,94].

With regards to post-amputation pain, Rauck et al. have shown that following two weeks of home trial nine responders reported reductions across several variables, including mean daily worst post-amputation pain, average residual limb pain, average phantom limb pain, residual limb pain interference, phantom limb pain interference, and Pain Disability Index up to four weeks following the end of stimulation. These positive findings were counterbalanced by minor decreases in the Beck Depression Inventory scores with little to no change in pain medication use.

Other approaches have also looked at peripheral nerve field stimulation (PNFS) where the electrode contact point is placed at the area of pain but not in direct contact with the nerve. The direct neural target using this form of peripheral field stimulation also targets Aβ nerve fibers consistent with peripheral nerve stimulation. Klomstein et al. evaluated the long-term efficacy and safety of PNFS in lower back pain in 105 patients at 1, 3, and 6 months post-implantation. They observed a stable decrease in pain at 6 months. Mean VAS score at baseline was VAS 7.9 (SD 1.38) and 4.7 (SD 1.99) at six-month follow up (*p* < 0.01). Statistically significant improvements were also seen across other parameters, including the Oswestry Disability Questionnaire, the Becks Depression Inventory, and the Short Form-12 item Health survey. Of the enrolled subjects 9.6% of the subjects experienced complications requiring surgical intervention [95]. Guentchev et al. also recently reported on the utility of PNS in managing sacroiliac joint (SIJ) pain [96]. This 12-patient study using eight pole electrode placed parallel to the SIJ joint showed at two weeks’ post-implant, subjects reported an average Oswetry Disability Index ODI reduction from 57% to 32% and VAS from 9 to 2.1. International Patient Satisfaction Index (IPSI) was 1.1. At six months, the mean ODI was 34% (*p* = 0.0006), VAS was 3.8 (*p* < 0.0001) and IPSI was 1.9. At 12 months, mean averages for 6 of 7 patients were ODI 21% (*p* < 0.0005), VAS 1.7 (*p* < 0.0001), and IPSI 1.3 [96].

With regards to headache and facial pain there has been numerous studies looking at the benefits for PNS on migraines. The ONSTIM study was a prospective single-blind 66 patient randomized study that showed a 39% response in the simulation group and 6% response in the pre-set simulation group based on an responder rate of greater than 50% or VAS improvement of 3 [97]. Dodick et al. presented 12-month data evaluating the use of PNS of the occipital nerves for patients with chronic migraine [98]. Headache days were significantly decreased by 6.7 (±8.4) days in the intent-to-treat (ITT) population (*p* < 0.001) and by 7.7 (±8.7) days in the intractable chronic migraine (ICM) population (*p* < 0.001). Excellent or good headache relief was also reported by almost two thirds of the ITT population and close to 70% of the ICM population. The study reported 183 device/procedure-related adverse events, of which 18 (8.6%) required hospitalization and 85 (40.7%) required surgical intervention [98]. Cluster headaches have been shown to respond to Sphenopalatine ganglion (SPG) stimulation as well stimulation of the pterygopalatine fossa [97].

There has also been good benefits reported from PNS for refractory sub-acromial impingement syndrome (SIS) [99], post-stroke shoulder pain [100], and post-traumatic brachial plexus trauma refractory to medical and surgical management [101].

Though an independent topic of its own right there has been considerable recent developments of implantable and portable vagus nerve stimulators that have shown to modulate nociception in addition to efficacy in treatment of refractory epilepsy and depression [102,103,104,105,106,107]. Clinical areas that have shown good effect have been trigeminal allodynia, fibromyalgia, chronic pelvic pain and headaches.

Several more recent advances in peripheral nerve stimulation technology has resulted in more improved compliance and ease of use. Deer et al. conducted an eight-patient trial targeting the median nerve for alleviating neuropathic pain using a novel stimrouter system with wireless battery to lead connectivity. They observed both pain reduction throughout the 5-day treatment period and reduced oral opioid consumption with no significant or unexpected adverse events [108]. This was followed by Deer et al. publishing a randomized double-blinded multicenter trial of 147 patients that showed that patients receiving active stimulation achieved a statistically significantly higher response rate of 38% vs. the 10% rate found in the Control group (*p* = 0.0048). Specifically, the treatment group achieved a mean pain reduction of 27.2% from Baseline to Month 3 of follow-up compared to a 2.3% reduction in the Control group (*p* < 0.0001). The study did not report any adverse events [109].

Potential reported adverse events mainly included lead migration, hardware issues (i.e., battery failure, lead of extension disconnection, programmer malfunction, IPG migration and malfunction). Other reported events include subcutaneous hematomas, seromas, skin erosions, pain and numbness at the IPG site, allergic reactions to surgical material, headache and muscle cramping [98,101,108,109,110].

## 3. Discussion

We have attempted to present a comprehensive review of the current areas of neuromodulation advances and their potential uses in various chronic pain pathologies. Spinal cord stimulation (SCS) is a well established modality to effectively control the pain of neuropathic origin. Its efficacy and safety has been demonstrated in several randomized controlled trials. For several decades conventional SCS was the only stimulation paradigm available to patients. Even though treatment with this modality showed great results, it was not without its shortcomings including but not limited to failure of therapy, unwanted paresthesias, and development of tolerance. In the last few years, research was focused on manipulation of SCS parameters to meet the physiological needs of the patients. Development of Burst SCS program was a step in this direction where stimulation mimics the natural neuronal firing patterns. It was found to be more effective than conventional SCS in the treatment of low back pain. Similarly, development of high frequency stimulation therapy, which is presumed to act via induction of depolarization blockade/desynchronization of neuronal signals has also shown superiority over conventional SCS in the management of chronic low back pain. These two new stimulation paradigms also provide patients with an option of paresthesia free stimulation, which may be preferred by some patients. However, the more recent research in the field of spinal cord stimulation is focused on altering the therapy to individual needs. Development of closed loop SCS is a step in this direction to mitigate the effects of positional changes and development of tolerance to the therapy.

While dorsal column stimulation has shown great promise, alternative technology outside the dorsal column focused on concentrating current in the dorsal root ganglion or targeting individual nerves as demonstrated through peripheral nerve stimulation has shown growing promise. Questions related to reduced energy dispersion, focused targeted therapy, and potential effects of these various dorsal column and peripheral nerve stimulator on the neuroimmune axis presents exciting future research. In addition, given the growing understanding of various waveforms and their respective effects on the medial and lateral pain pathways may provide more insight into mechanism of action and help to tailor more appropriate therapy for each individual patient.

While constant efforts are being made to advance the field of neuromodulation, a challenge that is consistently faced by researchers is the inability to produce ideal study designs. Secondary to the intrinsic nature of therapy, it is nearly impossible to blind the patient, physician, and the programmer to produce reliable test results. Also, use of “sham-effect” raises the ethical concerns of subjecting the patient to the risks of an interventional pain procedure with no benefit.

## 4. Conclusions

These are times of advancement in the field of bioelectrical medicine. With this progress comes new responsibilities for those involved in this revolution. The responsibilities include a commitment to improving efficacy, mitigating complications, and finding new innovations that may continue to evolve the progress that has been made to date. This should be done with a commitment to ethics and patient safety, and with a curiosity that inspires new ideas and discoveries.

## Figures and Tables

**Table 1:** Indications and outcomes of conventional SCS.

Study	Indication	Study Design	Methods	Outcome Measures	Results	Conclusion
Kemler et al. [19]	CRPS	Randomized trial	CRPS patients assigned in a 2:1 ratio to SCS + PT group (*n* = 36) & PT group (*n* = 18). 24 of 36 patients underwent permanent implant of SCS device.	VAS, GPE, functional status, health-related quality of life	Intention-to-treat analysis showed significant reductions in pain at 6 m in SCS + PT group (*p* < 0.001). Improvements in GPE also observed in SCS group.	SCS can reduce pain in carefully selected CRPS patients.
Harke et al. [20]	Sympathetically maintained CRPS	Prospective trial	CRPS patients underwent SCS implant, and pain intensity was estimated during SCS free intervals of 45 min every 3 m vs. under treatment.	VAS, pain disability index, reduction in pain medication	Improvements in VAS during treatment vs. SCS free intervals (*p* < 0.01). Reduction in pain meds during treatment (*p* < 0.01).	Functional status, quality of life, and pain medication usage can be improved with use of SCS in sympathetically mediated CRPS.
North et al. [21]	FBSS	Randomized controlled trial	50 FBSS patients randomized to SCS and reoperation. If results of randomized treatment unsatisfactory, patient could crossover to alternative.	Self-reported pain relief, patient satisfaction, crossover to alternative procedure	Among 45 patients available for follow up, SCS (9 of 19) was more successful than reoperation (3 of 26 patients) (*p* < 0.01). (5 of 24 in SCS group) vs. (14 of 26 in reoperation group) crossed over (*p* = 0.02).	SCS is more effective than reoperation in patients with persistent radicular pain after spine surgery.
Kumar et al. [22]	FBSS/Neuropathic limb pain	Multicenter randomized controlled trial	100 FBSS patients with predominant leg pain of neuropathic radicular origin randomized to SCS + CMM group vs. CMM alone group and followed for 6 m.	1^0^ outcome—≥50% pain relief in the legs. 2^0^ outcome—improvement in back and leg pain, health-related quality of life, functional capacity, use of pain medications	In the intention-to-treat analysis at 6m, 48% SCS patients (*n* = 24) & 9% CMM patients (*n* = 4) achieved 1^0^ outcome. SCS + CMM group also achieved the 2^0^ outcomes significantly more than the CMM alone group (*p* < 0.05 for all comparisons).	SCS is superior to CMM in the treatment of limb pain of neuropathic origin in patients with prior lumbosacral surgery.
De vos et al. [23]	PDN	Multicenter randomized controlled trial	60 PDN patients refractory to conventional medical therapy were randomized in 2:1 ratio to best conventional medical practice (with SCS) or without (control) SCS group and followed at regular intervals.	EuroQoL 5D, SF-MPQ, VAS	At 6m follow up, average VAS decreased from 73 (baseline) to 31 in SCS group (*p* < 0.001); VAS remained unchanged at 67 in control group (*p* = 0.97). SF-MPQ and EuroQoL 5D also improved significantly in the SCS group.	SCS therapy significantly reduced pain and improved quality of life in patients with PDN.
Van beek et al. [24]	PDN	Prospective two-center clinical trial	48 patients with PDN were treated with SCS and followed for 5 years.	NRS score for pain, PGIC, and treatment success (50% reduction of NRS score or significant PGIC)	Patients showed significant improvements in all outcome measures at the follow-up visits. Treatment success was observed in 55% of patients after 5 years, and 80% of patients with permanent implant continued to use their SCS device.	SCS is successful in alleviating pain in patients with PDN.

SCS—Spinal Cord Stimulation, VAS—Visual analog scale, GPE—Global perceived effect, PDN—Painful diabetic neuropathy SF-MPQ—Short-form Mcgill pain questionnaire, NRS—Numeric rating scale, PGIC—Patient’s global impression of change, PT—Physical Therapy, EuroQol 5D- EuroQol five dimensions questionnaire.

**Table 2:** Comparative studies between conventional/tonic & High Frequency (10 khz) SCS.

Study	Indication	Study Design	Methods	Outcome Measures	Results	Conclusion
Kapural et al. [26]	Chronic Intractable back and leg pain	Randomized controlled trial	198 subjects with back and leg pain, randomized in 1:1 ratio to HF SCS (>10 khz) or conventional SCS. Of these 171 passed trial and received permanent implant.	1^0^ outcome—≥50% pain relief in the back	At 3 months, In HF group 84.5% were responders for back pain (vs. 43.8% for tonic SCS) and 83.1% were responders for leg pain (vs. 55.5% for tonic SCS); (*p* < 0.001). Superiority of HF stimulation was sustained through a 12-month period.	HF stimulation was better than tonic stimulation for treatment of chronic intractable back and leg pain.
Kapural et al. [31]	Chronic Intractable back and leg pain	Randomized controlled trial	198 subjects with back and leg pain, randomized in 1:1 ratio to HF SCS (>10 khz) or conventional SCS. Of these 171 passed the trial and received permanent implant.	1^0^ outcome—≥50% pain relief in the back	At 24-months follow up, more subjects continued to be responders to HF stimulation than conventional SCS (back pain-76.5% vs. 49.3%, leg pain-72.9% vs. 49.3%; *p* < 0.001). Also back and leg pain decreased to a greater degree with HF stimulation than tonic SCS (*p* < 0.001).	HF (10 khz) stimulation was better than tonic stimulation for treatment of chronic intractable back and leg pain.
Amirdelfan et al. [32]	Chronic Intractable back and leg pain	Randomized controlled trial	198 subjects with back and leg pain, randomized in 1:1 ratio to HF SCS (>10 khz) or conventional SCS. Of these 171 passed the trial and received permanent implant. QOL and functional measures were collected up to 12 months.	ODI, GAF, CGIC, PSQI, SF-MPQ-2	At 12 months follow up; ODI-69.6% subjects were classified into lower disability category with HF (vs. 55.1% with tonic SCS; *p* = 0.01). Subjects had a more significant improvement in GAF scores in HF group vs. tonic SCS (14 vs. 6.5, respectively; *p* < 0.01). Significant improvements were seen in continuous, intermittent, and neuropathic pain in HF group vs. tonic SCS on the SF-MPQ-2 scale. However, no difference was observed on the affective disorders subscale. Significant improvements were also seen in the HF group on CGIC and PSQ1 scales compared to tonic SCS.	High frequency (10 khz) stimulation was better than tonic stimulation in improving quality of life and functional outcomes in patients with chronic intractable back and leg pain.

SCS—Spinal Cord Stimulation, High frequency—HF, ODI—Oswestry disability index, GAF—Global assessment of functioning, CGIC—Clinical global impression of change, PSQI—Pittsburgh sleep quality index, SF-MPQ—Short-form Mcgill pain questionnaire, QOL—Quality of life.

**Table 3:** Comparative studies between conventional/tonic & burst SCS.

Study	Indication	Study Design	Methods	Outcome Measures	Results	Conclusion
De ridder et al. [38]	Intractable neuropathy/FBSS/diabetic neuropathy	Retrospective analysis	Retrospective analysis of 102 patients who previously received SCS was performed. These were divided into two groups—the first group included patients who became failures to tonic stimulation and others who continue to respond. Both groups switched to burst SCS and followed up.	NRS pain scores, amount of responders.	It was reported that almost 25% of the patients were non-responders to conventional SCS and out of that, 63% responded to burst. Also, 95% who responded to tonic stimulation reported further improvement with burst SCS.	Burst was better than tonic stimulation and can also rescue non- responders.
Deer et al. [39]	FBSS/Persistent radicular pain	Randomized controlled trial	100 patients with a successful trial with tonic SCS randomized to receive tonic vs. burst stimulation for the first 12 weeks and the other stimulation mode for the next 12 weeks. Subjects then used the stimulation mode of their choice and were followed for a year.	1^0^ endpoint- assessment of VAS score (tonic vs. burst)	Intention-to-treat analysis was used to estimate the difference in overall VAS scores, which showed burst was superior to tonic stimulation (*p* < 0.017). Significantly more subjects (70.8%) preferred burst over tonic stimulation (*p* < 0.001).	Burst stimulation was superior to tonic stimulation for the treatment of chronic pain.
Demartini et al. [42]	FBSS/persistent radicular pain	Multicenter observational study	23 patients underwent 2 weeks of tonic stimulation followed by 2 weeks of burst stimulation.	1^0^ outcome-reduction of pain in the back and the legs. 2^0^ EuroQol-5D, PCS	Tonic stimulation reduced leg pain (*p* < 0.05), the burst mode added an extra pain reduction (ΔNRS 1.2 ± 1.5) (*p* < 0.01). Both stimulation paradigms failed to reduce back pain (*p* = 0.29) Secondary outcomes were achieved with both stimulation paradigms.	Burst stimulation was more successful than tonic stimulation in the treatment of leg pain.

SCS—Spinal Cord Stimulation, NRS—Numeric rating scale, VAS—Visual analog scale, PCS—Pain catastrophizing scale.

**Table 4:** Evidence for efficacy for DRG stimulation.

Study	Indication	Study Design	Methods	Outcome Measures	Results	Conclusion
Deer et al. [56]	Chronic intractable neuropathic pain of trunk and/or limbs	Prospective, multicenter, single arm, pilot study	10 subjects underwent trial with Dorsal root ganglion stimulation device and were followed up for 3–7 days.	Daily VAS scores, perceived % of pain relief at the final visit	Average pain reduction between baseline and final follow up visit was 70 + 32% (*p* = 0.0007). All subjects achieved pain relief in the desired specific regions of the body.	Authors concluded DRG could be a viable target for neurostimulation for the treatment of chronic intractable pain.
Liem et al. [72]	Chronic intractable neuropathic pain of trunk/limb and/or sacral region	Prospective, Multicenter study	32 subjects with successful trial with DRG stimulation underwent permanent implantation of the device. Patients were followed up for 6 months.	VAS, % of pain relief at follow up, improvements in quality of life (EQ-5D), mood, function.	At 6 month follow up, overall pain reduction was 56%; 52% patients had >50% pain relief. Improvements were seen in all other outcome measures.	Neuromodulation of DRG was effective in the treatment of chronic intractable neuropathic pain conditions. It is able to provide paresthesia coverage in areas such as foot, which were difficult to treat with traditional SCS.
Deer et al. [73]	CRPS	Multicenter, randomized controlled trial	152 subjects randomized in a 1:1 ratio to receive DRG stimulation vs. traditional SCS and were followed up at 3, 6, 9, and 12 months	1^0^ end point—>50% reduction in VAS scores at 3 month follow up 2^0^ end point- positional effects on paresthesia intensity	The percentage of subjects with >50% pain relief was greater in DRG arm (81.2%) vs. SCS arm (55.7%, *p* < 0.001) at 3 months. Subjects in DRG arm reported less postural variation in paresthesia (*p* < 0.001).	DRG stimulation was more effective and provided less postural variation as compared to conventional SCS.

DRG—Dorsal root ganglion, SCS—Spinal cord stimulation, VAS—Visual analog scale, EQ-5D—EuroQol five dimensions questionnaire.
